# Green Deep Eutectic Solvents for Functionalizing Chitosan–Dialdehyde Materials with Varied Crosslinker Content

**DOI:** 10.3390/ma19030529

**Published:** 2026-01-29

**Authors:** Magdalena Gierszewska, Ewa Olewnik-Kruszkowska, Kornelia Kadac-Czapska, Małgorzata Grembecka, Eliza Knez

**Affiliations:** 1 Department of Physical Chemistry and Physicochemistry of Polymers, Faculty of Chemistry, Nicolaus Copernicus University in Toruń, 87-100 Toruń, Poland; mgd@umk.pl; 2 Department of Bromatology, Faculty of Pharmacy, Medical University of Gdańsk, 80-416 Gdańsk, Poland; malgorzata.grembecka@gumed.edu.pl (M.G.); eliza.knez@gumed.edu.pl (E.K.)

**Keywords:** chitosan, potential food packaging, crosslinking, dialdehyde starch, deep eutectic solvent, plasticizer, molecular structure, mechanical properties, wettability

## Abstract

**Highlights:**

**What are the main findings?**

- The combined use of DAS and DES enabled the generation of a balance between stiffness and flexibility.
- Incorporation of deep eutectic solvent into the DAS-crosslinked chitosan causes a substantial increase in surface roughness.
- DES markedly lowers the water contact angle, enhancing surface hydrophilicity beyond roughness-driven effects.

**What are the implications of the main findings?**

- DES plasticization enables more flexible Ch/DAS films and easier processing.
- An optimized DAS amount in Ch/DES films ensures a balanced compromise between stiffness, strength, and flexibility.
- Lower water CA implies higher polarity at the interface, aiding food coating creation.

**Abstract:**

A series of chitosan-based films was obtained by combining the covalent crosslinking of chitosan with dialdehyde starch (DAS) and plasticization using a choline chloride–malonic acid deep eutectic solvent (DES), thereby engineering their structural, mechanical, and surface properties for advanced packaging applications. DAS was synthesized via periodate oxidation of potato starch and characterized by FTIR and quantification of aldehyde groups through acid–base titration, enabling precise control of the –NH_2_ (chitosan) to –CHO (DAS) molar ratios (40:1, 20:1, 10:1) used for film formation. Chitosan films (neat, DAS-crosslinked, DES-plasticized, and DES-plasticized/DAS-crosslinked) were obtained by solution casting, with constant total chitosan and/or Ch+DES mass across formulations, and subsequently examined in terms of molecular structure, density, mechanical characteristics, micro- and nanoscale morphology, color, wettability, and surface free energy. The most significant changes relevant to potential applications were observed in mechanical properties and surface free energy. The incorporation of DAS and DES into chitosan resulted in a significant reduction in Young’s modulus from 1150 MPa to 130 MPa, accompanied by a significant increase in elongation at break—from 10% to almost 90%. Moreover, it should be noticed that the addition of DAS and DES led to a nearly twofold increase in surface free energy, from 32.5 to 59.9 mJ m^−2^. While previous studies have predominantly focused on single modifications of chitosan—either covalent crosslinking with dialdehyde starch (DAS) or plasticization with deep eutectic solvents (DES)—this work introduces a pioneering dual-modification strategy that simultaneously integrates both techniques, representing the first systematic investigation of their synergistic effects unattainable through individual approaches.

## 1. Introduction

It is known that plastics constitute the most considerable tonnage of municipal solid waste, with a significant percentage used for food packaging purposes [1]. The strategic shift toward minimizing polymer food-packaging waste involves a systemic reorganization driven by stringent regulatory mandates, material innovation, and redesign of consumption models, focusing equally on preventing waste generation and maximizing end-of-life recovery [2]. To achieve the aforementioned goals, modification of existing synthetic polymers, while simultaneously substituting them with alternative materials, is proposed.

Even if the market for bio-based and biodegradable polymers is expanding rapidly, the replacement of petroleum-based plastics is limited, mostly due to their performance and large-scale manufacturability for biodegradable substrates [3,4]. As such, the current trend in the development of food-packaging materials focuses on various strategies to enhance the chosen physicochemical features of biodegradable polymer-based packaging. Yao et al. [5] reported a strong growth of polysaccharide-based packaging. Polysaccharides have attracted particular attention due to their renewability, low toxicity, and high amenability to chemical functionalization. Within this group, chitosan stands out for its intrinsic bioactivity and strong film-forming ability, making it a promising platform for the development of functional packaging materials [6]. Much research has already evaluated chitosan-based films and edible coatings directly on diverse food matrices, consistently confirming their ability to preserve quality and extend shelf-life through barrier effects and antimicrobial/antioxidant functionality [7,8].

Chitosan (Ch) is a linear, cationic copolymer obtained primarily via the alkaline N-deacetylation of chitin. At the molecular level, it is a copolymer of β-(1→4)-linked D-glucosamine and N-acetyl-D-glucosamine units, with the “chitosan” term typically referring to materials containing >60% D-glucosamine residues [9]. Ch is insoluble in water and common organic solvents but exhibits film-forming properties. This feature is extremely important for creating a food-coating layer. As stated in the literature, such coatings are smooth, transparent, slightly yellowish [8,10], and thus accepted by customers. Moreover, it has been found that Ch possesses high sorption capacity and bactericidal properties, making it a promising packaging material [6,11]. The other important properties of chitosan, making it an interesting choice for food-packaging applications, are its ease of further enhancing its antimicrobial and antioxidative properties, resulting in an “active packaging” formulation [8,12]. When used as an edible coating, chitosan forms a semi-permeable surface layer that can regulate O_2_/CO_2_ exchange and fruit respiration/metabolism, thereby delaying senescence and improving postharvest quality [13]. In other cases, chitosan film reduces O_2_ permeation and thus prevents, e.g., lipid oxidation or dye degradation [14]. However, structural factors may compromise the mechanical performance of chitosan-based materials, influencing also other functional properties. Neat Ch films are brittle and suffer from a lack of elasticity [15]; thus, reinforcement of the material is needed for targeted material design. To overcome these drawbacks, multiple modification routes are routinely applied, including crosslinking, enzymatic treatment, graft copolymerization, complexation/polyelectrolyte complex formation, filler incorporation, and blending with other biopolymers or compatible polymers to improve mechanical integrity. A representative example is chemical crosslinking, which can increase mechanical strength and water resistance compared to uncrosslinked chitosan films. A large number of compounds have been tested as chemical chitosan crosslinkers, with aldehydes among the most well-known and widely used group; however, many synthetic crosslinking agents are harmful to both humans and the environment [16,17]. To fully follow the initial aim of developing chitosan-based materials, the crosslinker should also be non-toxic and biodegradable. Moreover, because chitosan can form an edible coating, it is worth considering an edible crosslinker. Dialdehyde starch meets these assumptions. The oxidized starch (E1404), known as a starch with hydroxyl groups oxidized to carboxyl or aldehyde groups, is legally used in foods in the EU and has been safety-reviewed. E1404 can be eaten as a starch-derived additive when used within regulations and good manufacturing practice [18].

Dialdehyde starch (DAS) is obtained through the controlled oxidation of starch hydroxyl groups to aldehyde groups. DAS is a reactive biopolymer capable of forming covalent bonds with the nucleophilic amino groups of Ch. The resulting –C=N– bonds enable the formation of crosslinked biocomposites, in which Ch acts as the cationic component, and DAS serves as the reactive crosslinking agent and modifier of rheological and mechanical properties. Besides being a “green” substitute for common aldehyde crosslinkers, DAS also serves as a platform for active packaging, as Schiff-base structuring and/or DAS–bioactive conjugates can reduce light/UV transmittance and deliver antibacterial activity, thereby directly helping suppress photo-oxidation and microbial spoilage in food systems [19]. Research on modified starch composites suggests that elasticity, dimensional stability, and resistance to water migration can be tailored by adjusting the degree of oxidation and processing conditions [20]. Further incorporation of plasticizers can significantly impact the elasticity of such materials [21]. In this context, deep eutectic solvents (DESs), described as “green chemical compounds”, which are non-toxic and biodegradable substances whose application in biopolymer processing supports the principles of green chemistry and sustainable development, are of particular interest.

Deep eutectic solvents are mixtures of two or more compounds capable of associating with each other through hydrogen-bond complexation (e.g., choline chloride and maleic acid, ChCl:MA). They are characterized by a depressed melting point relative to the starting components, high solvent capacity for many organic and inorganic compounds, tunable polarity and rheological properties, and low volatility [22,23]. In recent years, it has been shown that DESs can act not only as a solvent but also as a modifier of interfacial interactions, a plasticizer and an active additive, imparting new properties to materials. In recent years, in Ch-based systems, DESs have been used not only as a plasticizers, but also as a medium for polymer chemical modification, for film preparation, and as carriers of natural bioactive compounds [24,25]. Ciocirlan et al. [26] developed chitosan films plasticized with a choline chloride:glycolic acid DESs (also used as the extraction medium for hawthorn polyphenols) and showed that DES-driven hydrogen-bonding plasticization improves flexibility while the co-delivered polyphenols enhance UV-barrier and antioxidant performance, supporting DES-enabled active food-packaging concepts. Rolińska et al. [27] prepared chitosan films incorporating choline chloride-based DESs (with different hydrogen-bond donors) and reported that a ChCl:citric acid DES formulation delayed bread mold spotting by up to 29 days versus polyethylene while maintaining mechanical performance in the PE-relevant range. Alasalvar et al. [28] extracted *Hibiscus sabdariffa* bioactives using choline chloride–carboxylic-acid DESs (with ChCl–oxalic acid providing the highest extraction yield) and subsequently incorporated selected DESs and DES-based extracts into chitosan films as plasticizers; the resulting formulations enabled a tunable strength–flexibility trade-off, with an outstanding light-barrier reported particularly for the ChCl–tartaric acid DES, while the DES-extracts delivered markedly higher antioxidant activity than the neat DES counterparts. In turn, Lawal et al. [29] incorporated a DES-derived date-seed polyphenolic extract (1.5–3%) into chitosan–PVA films and showed that the DES-enabled extract acts as a functional additive that boosts antioxidant/antibacterial and UV-shielding properties and measurably slows refrigerated shrimp quality deterioration compared with conventional cling film.

Despite the dynamic development of research on DESs and polysaccharide composites, relatively few studies focus on systems combining Ch with DAS, in which DESs function simultaneously as a processing medium and as a means of controlling material properties. Therefore, the aim of this work is not only to examine how the concentration of DAS affects the structural, mechanical, and surface properties of chitosan films, but also to assess the effect of DES plasticizer presence. The effect of introducing plasticizer into DAS-crosslinked chitosan films on selected parameters relevant to potential packaging applications was aimed to be evaluated. The realization of this goal was carried out according to a systematic research plan involving controlled variations in system composition, reproducible processing conditions, and a comprehensive analysis of the results. Obtaining such information is essential for the rational design of new materials with high application potential. The results obtained in this study will help fill the existing research gap and support the further development of new biopolymers.

## 2. Materials and Methods

### 2.1. Materials

The chitosan was purchased in powder form (Ch, degree of deacetylation DDA = 83.4 ± 2.4%, average molecular weight 157 ± 17 kDa [30] from BioLog Heppe GmbH (Landsberg, Germany). Potato starch and sodium periodate (NaIO_4_, analytical grade) were obtained from Chempur (Piekary Śląskie, Poland). For the characterization of dialdehyde starch (DAS), standardized solutions of sodium hydroxide (0.25 M) and hydrochloric acid (0.25 M) (Chempur (Poland)) were applied. Deep eutectic solvents were prepared from choline chloride (ChCl, 99%) supplied by Acros Organics (Geel, Belgium) and malonic acid (MA, 99%, analytical grade) purchased from Sigma-Aldrich (Hamburg, Germany). Other reagents included acetic acid (99.5–99.9%, analytical grade, Avantor S.A., Gliwice, Poland), acetone (analytical grade, Stanlab, Lublin, Poland), diiodomethane (99%, stabilized, Alfa Aesar, Karlsruhe, Germany), and anhydrous glycerol (analytical grade, Avantor S.A., Poland). All reagents were used without further purification. Deionized water (resistivity ≥ 18.2 MΩ cm) was used for all preparations.

### 2.2. Starch Oxidation

Dialdehyde starch (DAS) was obtained by oxidizing potato starch with a sodium periodate solution, following the method described by Lu et al. [31] with some modifications. An amount of 3 g of potato starch was weighed, quantitatively transferred into a 250 mL three-necked flask, and suspended in 60 mL of distilled water. The mixture was then stirred at 40 °C. Then, a known amount of sodium periodate solution (0.28 M) was added dropwise to achieve a 2:1 weight ratio of starch to sodium periodate. The modification was carried out at a constant temperature of 40 ± 1 °C for 2 h. Finally, acetone was added, and the resultant product, appearing as a fluffy precipitate, was filtered, washed with a fresh portion of acetone, air-dried in the shade for 24 h, and then vacuum-dried for another 24 h at 25 °C. The final product was stored in a light-impermeable, tightly sealed container at 4 °C for further use.

### 2.3. Dialdehyde Starch Characterization

To confirm the effectiveness of the modification process, the Fourier-transform infrared (FTIR) spectra of native and oxidized starch have been recorded in KBr disk form, scanning the sample from 400 to 4000 cm^−1^ with 2 cm^−1^ resolution using a Nicolet iS10 spectrometer (Thermo Fisher Scientific, Waltham, MA, USA).

The aldehyde group content in the obtained dialdehyde starch (DAS) was quantified by acid–base titration according to the method proposed by Hofreiter et al. [32]. Approximately 0.1 g of DAS was weighed, then 5 mL of a standardized 0.25 M NaOH solution was added. The flask was placed in a water bath at 70 °C for 2 min to ensure complete dissolution of the sample, followed by rapid cooling to room temperature. Then, 7.5 mL of a standardized 0.25 M HCl solution and 15 mL of distilled water were added to the flask. The mixture was titrated with a standardized 0.25 M NaOH solution using phenolphthalein as an indicator. All measurements were performed in triplicate. The percentage of dialdehyde units was calculated according to the equation:

(1) $$\%\;aldehyde\;group\;content=\frac{n_{CHO}}{n_{glucose\;units}}=\frac{C_{NaOH}\cdot V_{NaOH}-C_{HCl}\cdot V_{HCl}}{(m_{DAS}/161)\cdot 1000}\cdot 100\%,$$

where *n_CHO_*—moles of aldehyde groups [mol], *C*—concentration of NaOH or HCl [mol·L^−1^], *V*—total volume of NaOH or HCl solution used [mL], *m_DAS_*—the mass of DAS [g], and 161—represents the molecular weight of the repeating unit in starch, 50% of which has been converted to dialdehyde units [g·mol^−1^].

### 2.4. Chitosan Films Formation

Prior to forming crosslinked and/or plasticized films, chitosan and dialdehyde starch solutions were prepared. Then, 1% (*w*/*v*) chitosan solution was obtained by dissolving a known mass of Ch in 2% (*w*/*v*) CH_3_COOH. DAS was dispersed in water and then heated at 60 °C until a clear, transparent solution of 10 g·L^−1^ was reached. A deep eutectic solvent (DES) was prepared by mixing choline chloride and malonic acid in a 1:1 molar ratio, followed by heating the mixture under stirring at 65 °C until a homogeneous liquid was formed.

First, DAS-crosslinked chitosan films were prepared by mixing chitosan and DAS solutions in the ratio providing the given molar ratio of amino groups of chitosan (–NH_2_, based on DDA of chitosan) to the aldehyde groups of DAS (–CHO, based on aldehyde groups content) equal 40:1, 20:1, and 10:1. The mixtures were casted on a clean glass plates, evaporated to dryness at 25 °C, and finally dried for 24 h under reduced pressure to remove residual solvent. The uncrosslinked chitosan film was also obtained as a reference. Next, an analogous series of DES-plasticized uncrosslinked, and DAS-crosslinked chitosan films was prepared. The known volume of chitosan solution was first mixed with liquid DES and stirred for 12 h. A 50 wt.% DES content in relation to Ch was applied to all plasticized preparations. Then, the DAS solution was introduced, providing 40:1, 20:1, and 10:1 –NH_2_ to –CHO molar ratios. The films were formed as before. The uncrosslinked chitosan–DES film was also obtained for comparison. All prepared films had the same surface and an identical total mass of Ch and DES. DAS-crosslinked samples of a given -NH_2_:–CHO (a:b) ratio are denoted as Ch/DAS-a:b, while their DES-containing equivalents as Ch-DES/DAS-a:b.

Before further testing, the thickness of all films was determined using a Sylvac S229 thickness gauge (Crissier, Switzerland), with 0.001 mm accuracy. The measurements were taken at 20 randomly distributed points and averaged.

### 2.5. Polymeric Films Density

The influence of the crosslinking agent and the plasticizer on the density of the obtained chitosan films was evaluated by determining their mass and geometrically derived volume. Circular specimens with a diameter of *R* = 25.00 mm were prepared using a manual press and a steel punch. The thickness (*h*) of each sample was measured with Sylvac S229 thickness gauge (Switzerland). Assuming a cylindrical geometry and knowing the sample mass (*m*), the film’s density (*d*) was calculated using the formula:

(2) $$d=m/(\pi \cdot {\left(R/2\right)}^{2}\cdot h)\;[g\cdot {cm}^{-3}].$$

### 2.6. Molecular Structure of Films by Fourier-Transformed Infrared Spectroscopy

The chemical structure of the fabricated chitosan-based films and the effects induced by the plasticizer and the crosslinking agent were elucidated using Fourier-transform infrared spectroscopy (FTIR). FTIR spectra were recorded using the attenuated total reflectance (ATR) technique in the mid-infrared region (4000–400 cm^−1^) with a Bruker Vertex 70 spectrometer (Hamburg, Germany) equipped with a monolithic diamond ATR crystal with a 4 cm^−1^ resolution. The spectra of chitosan, dialdehyde starch powder, and DES liquid were used as references. For band intensity comparison, some spectra have been normalized, taking as a reference the band at 2924 cm^−1^, as it is known that among all fully resolved bands, the chosen one remains unaffected by polymer processing.

### 2.7. Mechanical Properties

The mechanical properties of the chitosan-based films were evaluated using a Shimadzu EZ-SX 100N universal testing machine (Kyoto, Japan) in accordance with ISO 527-3:2018 [33]. For each film, 5 specimens were cut and subjected to uniaxial tensile testing at a crosshead speed of 1 cm·min^−1^. Elongation at break (ε), tensile strength (σ_m_), and Young’s modulus (E) were derived from recorded stress–strain curves.

### 2.8. Scanning Electron Microscopy Analysis

Scanning electron microscopy (SEM) was conducted using a LEO 1430 VP instrument (Leo Electron Microscopy Ltd., Cambridge, UK) to examine both surface and cross-sectional morphologies. Prior to imaging, dry film specimens were fractured in liquid nitrogen to obtain representative cross-sections. All samples were coated with a thin gold layer via sputtering to ensure adequate electrical conductivity during SEM analysis.

### 2.9. Atomic Force Microscopy

The surface topology and roughness of the thin films were examined under ambient conditions using a NanoScope MultiMode scanning probe microscope (Veeco Metrology Inc., Santa Barbara, CA, USA) operating in tapping mode. Square sections of the dried films (1 × 1 cm^2^) were excised prior to imaging. Roughness parameters, including the root-mean-square roughness (Rq, the square root of the mean of the squared height deviations from the same reference plane) and the arithmetic average roughness (Ra, the mean of the absolute height deviations relative to the central plane), were calculated using Nanoscope v6.11 analysis software (Bruker Optoc GmbH, Ettlingen, Germany) from scans acquired over a 10 × 10 µm^2^ surface area.

### 2.10. Color Analysis

Color alterations induced by the incorporation of DES and/or DAS into the chitosan films were assessed using a MICRO-COLOR II LMC 6 colorimeter (Dr. Lange, Berlin, Germany), which operates within the CIE Lab color space. Based on the measured lightness (*L*) and the chromaticity coordinates *a* (ranging from green (−) to red (+)) and *b* (ranging from yellow (+) to blue (−)), the total color difference (Δ*E*), chroma (*C*), yellowness index (*YI*), and browning index (*BI*) were calculated for each sample:

(3) $$\Delta E=\sqrt{{\left(L-L^{*}\right)}^{2}+{\left(a-a^{*}\right)}^{2}+{\left(b-b^{*}\right)}^{2}},$$

(4) $$C^{*}=\sqrt{a^{2}+b^{2}},$$

(5) YI=[142.86·b]/L,

(6) $$\begin{array}{c} BI=\left[100\left(x-0.31\right)\right]/0.17\\ x=(a+1.75L)/(5.645L+a-3.012b). \end{array}$$

where L*, a*, and b* represent the values of the reference chitosan film.

### 2.11. Surface Wettability

The wettability and surface hydrophilicity of the fabricated chitosan-based films were evaluated through static contact angle (CA) measurements using two probe liquids of different polarity: diiodomethane (non-polar, surface tension 50.8 mN m^−1^) and water (polar, surface tension 72.7 mN m^−1^). Measurements were carried out in accordance with ISO 8296:2003 [34] using a Theta Flex goniometer (Biolin Scientific, Gothenburg, Sweden) under controlled laboratory conditions (approximately 23 °C and 50% relative humidity, with a drop volume of 2 μL). Image J software (Image J, NIH—v. 1.53r freeware version) was used for data analysis with an accuracy of ±1°. Surface free energy was calculated using the Owens, Wendt, Rabel, and Kaelble (OWRK) method [35,36]. For each sample, the total surface free energy (SFE, $\gamma_{S}$), and its polar ($\gamma_{S}^{P}$) and dispersive ($\gamma_{S}^{D}$) components were determined from the arithmetic mean of ten independent measurements per test liquid.

### 2.12. Resistance to UVA and UVB

Optical properties of all tested materials were examined to assess their UV protection capabilities. Spectra were acquired in the 200–800 nm range using a Halo DB-20 spectrophotometer (Dynamica Scientific Ltd., Newport Pagnell, UK). UV-blocking performance was evaluated across UVA and UVB regions, employing the following equations:

(7) $${UVA}_{blocking}=100-T_{UVA},$$

(8) $${UVB}_{blocking}=100-T_{UVB},$$

where $T_{UVA}$ and $T_{UVB}$ represent the average transmittance value in the given range.

## 3. Results and Discussion

### 3.1. Starch Modification and Dialdehyde Starch Characterization

**Figure 1:**
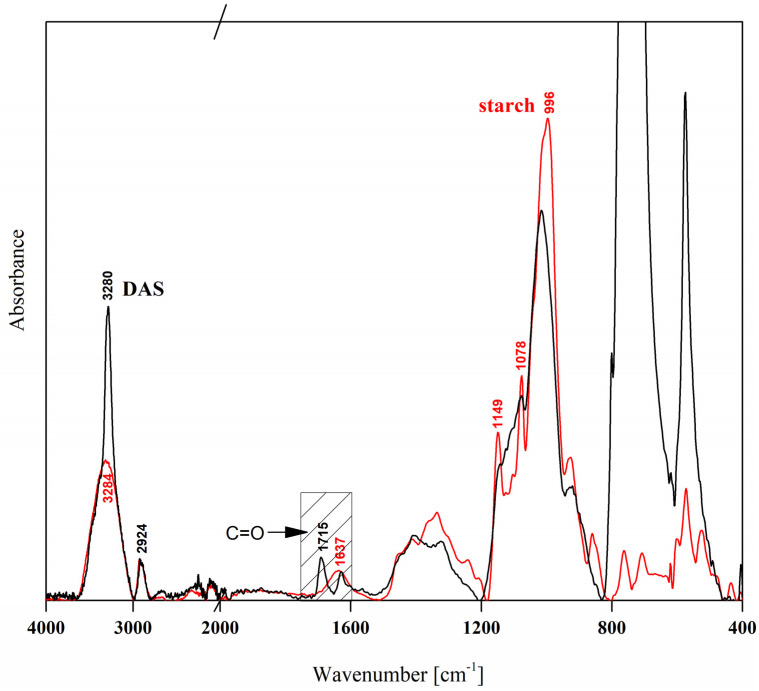
FTIR spectra of potato starch and its oxidized derivative.

In Figure 1, FTIR spectra of starch powder and its oxidized derivative are presented. Both spectra have been first baseline-corrected and then normalized, taking as a reference the band at 2924 cm^−1^, as it is known that among all fully resolved bands the chosen one stay unaffected upon oxidation process [37]. The starch spectra reveal the band characteristic for this polysaccharide [38,39,40], i.e., at 3284 cm^−1^ (stretching vibration of O–H groups), 2924 cm^−1^ (C-H stretching vibration), 1149 cm^−1^ (C-O-C stretching vibrations), 1078 cm^−1^ (C-C stretching vibrations), 996 cm^−1^ (pyranose ring vibration, C-O and C-C), and 860 cm^−1^ (axial or equatorial C-H bonds to the ring in pyranose sugar). The less intense band at 1637 cm^−1^ is associated with OH, attributed to the presence of bound water within the starch structure [38]. The above-mentioned vibrations reflect the α-D-glucopyranose units that are linked primarily by α-(1→4) glycosidic bonds, with additional α-(1→6) linkages at branching points, creating starch amylose and amylopectin macromolecules [41]. The treatment of potato starch with NaIO_4_ resulted in visible changes in the polysaccharide structure. The primary evidence of the oxidation process is a new band at 1715 cm^−1^, representing a C=O stretching vibration of aldehyde groups formed when NaIO_4_ selectively breaks the C2-C3 bond. Simultaneously, some changes in the bands characteristic of the native starch were noted, mostly visible as a loss of resolution of particular bands in a fingerprint region: (a) distinct reduction in intensity at 996 cm^−1^ resulting from opening of the glucopyranose ring, (b) reduction in the band intensity at 1149 cm^−1^, and (c) decreased intensity of the band at 1078 cm^−1^ caused by the ring opening process. Finally, the modification affected the shape (visibly narrowed) of the OH vibration band, indicating the breaking of hydrogen bonds. Similar changes in different extent were previously observed in the FTIR spectra of starches from different sources during the oxidation process [37,39,42,43] and used as a main proof of effective modification.

A classic acid–base titration was used to determine the content of aldehyde groups. The theory underlying this analysis is the Canizzaro reaction, i.e., the disproportionation of two aldehyde groups into an alcohol and a carboxylic group [32]. The found percentage of oxidized units, i.e., containing each two aldehyde groups, was (46.44 ± 0.78)%, which is consistent with the values given by others using a similar oxidation method [37,43]. As mentioned earlier, the final efficiency of the reaction is influenced by the proportion of starch and periodate, the type of starch, and the amylose content [37,43]. Typically, the modified starch still retains a certain percentage of unaffected mers. The aldehyde group content, together with the chitosan deacetylation degree, was used to calculate the reaction mixture composition, providing different molar ratios between the -NH_2_ groups of chitosan and the -CHO groups of DAS.

### 3.2. Molecular Structure of Ch/DAS and Ch-DES/DAS Films

**Figure 2:**
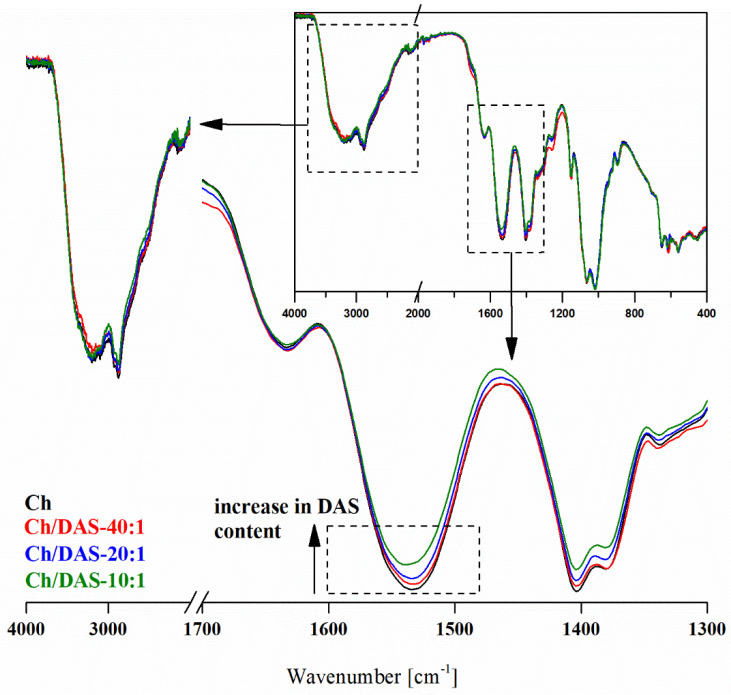
Effect of chitosan:dialdehyde starch ratio on the crosslinking efficiency as observed in FTIR normalized spectra of polymeric films.

In Figure S1 (Supplementary Materials) and Figure 2, FTIR spectra of neat chitosan film and films containing various amounts of dialdehyde starch are provided. In the FTIR spectra of chitosan, all bands characteristic of this polymer can be found, as we discussed earlier [44]. Among those substantial to the current discussion, the band at 1534 cm^−1^, corresponding to N–H bending in the amide group (amide II vibration), and the band at 1634 cm^−1^, representing C=O stretching in the amide group (amide I vibration), are visible in the Ch spectrum.

As both chitosan and starch macromolecules are structurally very similar, and the applied crosslinker amount is relatively low compared to chitosan, only discrete but still visible changes have been noted when comparing Ch and Ch/DAS samples (Figure S1, Supplementary Materials). The first evidence of the crosslinking reaction is the lack of the band characteristic to aldehyde groups of DAS in all Ch/DAS samples. Moreover, a decrease in intensity and a slight shift toward higher wavenumbers of the band representing the primary amine (approx. 1535 cm^−1^) were noticed (Figure 2). This indicates the change in the N-atom environment and the transition from a primary amine form to a secondary type. Both observations suggest that the reaction occurred between the -NH_2_ functionals of chitosan and the -CHO functionals of DAS, resulting in the formation of an imine (C=N) covalent bond between the two polymers. The formation of crosslinks could potentially also be confirmed by a new band at approximately 1630–1660 cm^−1^, characteristic of the C=N stretching vibration [45]. However, as also discussed by others [46,47], this band cannot be distinguished as it overlaps with the amide I vibration of chitosan.

There is also a visible effect of different ratios between chitosan and crosslinker on the FTIR spectra. As shown in Figure 2, the lower ratio of chitosan -NH_2_ functionals to the -CHO of DAS used, i.e., the higher crosslinker content, the more substantial reduction in intensity of amide II vibration band, and higher band shifting. As for the Ch/DAS-10:1 sample, which has the highest DAS content, no band representing C=O vibration in the aldehyde group was observed; it can be stated that within the applied crosslinking conditions, practically all -CHO groups undergo reaction with amino functionals. As a result, the number of chitosan/dialdehyde starch crosslinking bonds increases in series Ch < Ch/DAS-40:1 < Ch/DAS-20:1 < Ch/DAS-10:1. Similar variations in band intensities indicating higher crosslinking densities have also been observed for the Ch-DES/DAS samples crosslinked with different amounts of dialdehyde starch containing deep eutectic choline chloride:malonic acid mixture.

**Figure 3:**
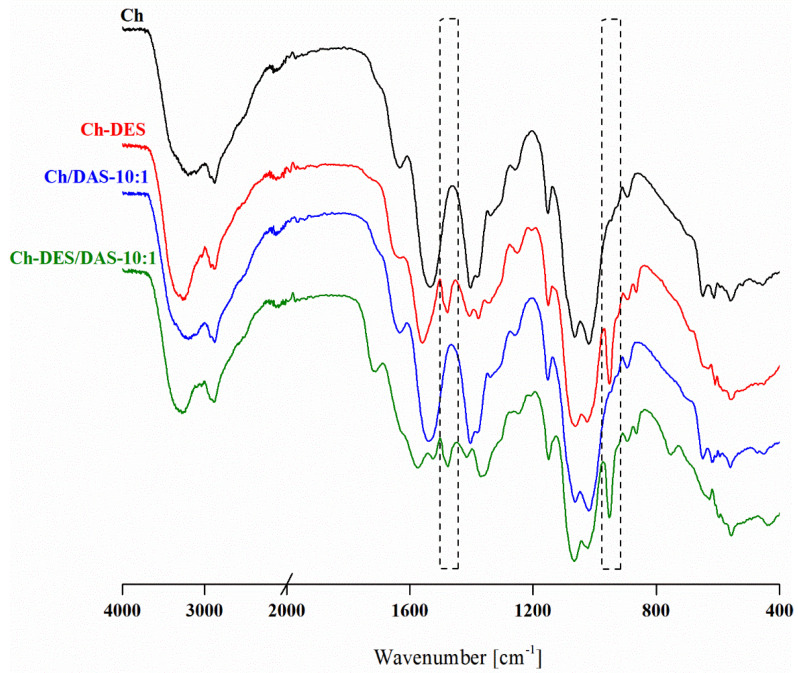
The FTIR spectra of neat Ch and Ch/DAS-10:1 samples before and after DES addition.

In Figure 3, the effect of the incorporation of choline chloride:malonic acid deep eutectic mixture into the chitosan and DAS-crosslinked chitosan matrix is given. To ensure clarity of analysis, the main peaks attributed to the DES have been marked with vertical rectangles. In both Ch-DES and Ch-DES/DAS-10:1 spectra, bands characteristic of neat chitosan and DAS-crosslinked chitosan can be noted; however, their position and shape have been affected by the presence of DESs. The narrowing and shifting towards higher wavenumbers of the O-H and N-H vibrational bands in the 3000–3700 cm^−1^ region confirm changes within the H-bond interactions. Specifically, the deep eutectic solvent disrupts existing H-bonds between chitosan chains and within the chitosan/dialdehyde starch polymeric network, while possibly forming new ones between the matrix and DES components. As has been shown by us [22,30] and others [48], at 50 wt.% DES the intensity of the amide I band is very low, confirming the engagement of amino groups in the interactions with DESs. Moreover, in the 1000–1150 cm^−1^ region, associated with C–O–C and C–OH vibrations of the chitosan pyranose rings, noticeable changes in bands relative intensity were observed upon DES incorporation. Since the ChCl:MA mixture also exhibits absorption in this region, these spectral changes cannot be attributed solely to hydrogen bond reorganization in the presence of DES, but should rather be explained as both overlapping contributions accompanied by a reorganization of hydrogen-bonding interactions within the DES-containing system. Additionally, the presence of DES in the polymeric material is confirmed by these DES bands, which do not overlap with those of the polymers, i.e., at approximately 1477 (N-H vibrations in quaternary ammonium groups of ChCl) and 952 cm^−1^ (C-N vibration in choline cation) (see DES spectrum, Figure S2, Supplementary Materials).

All the above-discussed differences and changes confirm the effectiveness of DAS crosslinking and higher crosslinking density for higher DAS applied. Simultaneously, the DES additive acts as a plasticizer, altering the H-bond interactions within the polymeric film.

### 3.3. Thickness and Density

The average thickness and density values of the neat chitosan and DAS-crosslinked and/or DES-containing chitosan films are summarized in Table 1.

As can be noted, all Ch/DAS and Ch-DES/DAS films had thicknesses similar to the Ch control sample (0.237 mm) and within the narrow 0.233–0.270 mm range. It can be seen that the addition of DAS to chitosan causes a slight increase in the *h* value. The average Ch/DAS film thickness reaches a maximum of approximately 0.245 mm for Ch/DAS-20:1, and further increasing the crosslinker content does not affect this value. This slight change can be attributed to the crosslinking of the Ch matrix by the starch dialdehyde derivative, as the crosslinks formed, confirmed by FTIR analysis, restrict the mobility of the chitosan chains. Similarly, the introduction of DES alone slightly increased the film thickness from 0.237 mm for Ch to 0.239 mm for the Ch-DES sample. As for Ch/DAS samples, also for the Ch-DES/DAS ones, the increase in DAS content causes a minor increase in the *h* value, noticeable especially for Ch-DES/DAS-10:1 (0.270 mm), which exhibited the greatest thickness among all films being examined. In this system, DES may increase polymer chain mobility, whereas a higher degree of crosslinking may partially stabilize the structure and limit shrinkage during the drying process. The minimal variation in thickness suggests that the application of DAS and ChCl-MA mixture does not significantly alter the coating formation process or the final film geometry.

Finally, no statistically significant differences were found between the thickness values of all obtained materials. The obtained results are consistent with literature reports indicating no significant changes in thickness after introducing various additives into the chitosan matrix [49,50].

The effect of different additives (DAS and/or DESs) on the density of the developed films has also been considered (Table 1). The density of the reference Ch film was found to be 1.403 g·cm^−3^. The addition of the DAS crosslinking agent at a 40:1 NH_2_:CHO molar ratio caused a decrease in density to 1.343 g·cm^−3^, which may result from insufficient crosslinking of the structure. It can be supposed that the disruption of existing H-bonds (loosening of the molecular structure) prevails over the crosslinking phenomenon. When a higher crosslinker amount was applied, the density of the material increased, reaching 1.442 g·cm^−3^ for the film with the highest DAS content (Ch/DAS-10:1). This behavior is consistent with the previous FTIR-analysis-based findings, confirming the increased crosslinking efficiency, and thus densification of the material structure, with the amount of crosslinker used. Conversely, the addition of ChCl-MA alone resulted in a decrease in film density to 1.264 g·cm^−3^. The effect of DES is based on the penetration of polar molecules between Ch chains, which weakens the H-bonding network and leads to loosening of the film structure. In the presence of both DESs and DAS, a complex and nonlinear effect on density was observed. For the sample containing both modifiers at a molar ratio of NH_2_:CHO = 10:1, the density reached the lowest value among all tested systems (1.214 g·cm^−3^).

### 3.4. Surface and Cross-Section Topography

**Figure 4:**
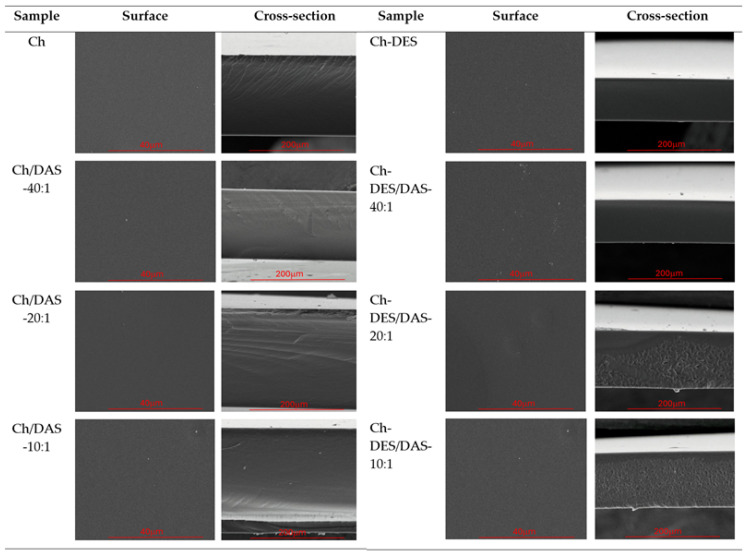
SEM images of Ch, Ch-DES, Ch/DAS, and Ch-DES/DAS films.

Figure 4 shows SEM images of both the surface and the cross-section of the studied films. The surface images, at the applied magnification (5000×), are free of cracks and appear to represent homogeneous materials. Thus, it can be stated that no phase separation or immiscibility of the components occurs in the investigated systems. Similarly, in the cross-sections, most films display a uniform, compact structure.

An exception is observed for the chitosan/DES-based materials containing higher amounts of dialdehyde starch (Ch-DES/DAS-20:1 and Ch-DES/DAS-10:1). For these two samples, the material no longer remains compact but forms an interpenetrated, porous structure. This effect was not observed in our other work [22], where chitosan-based materials containing various amounts of a DES composed of choline chloride and malonic acid were investigated. Therefore, this phenomenon can be clearly attributed to the increased starch content, which, by forming a covalent network with chitosan, may hinder the formation of a homogeneous mixture with DESs throughout the entire film volume.

**Figure 5:**
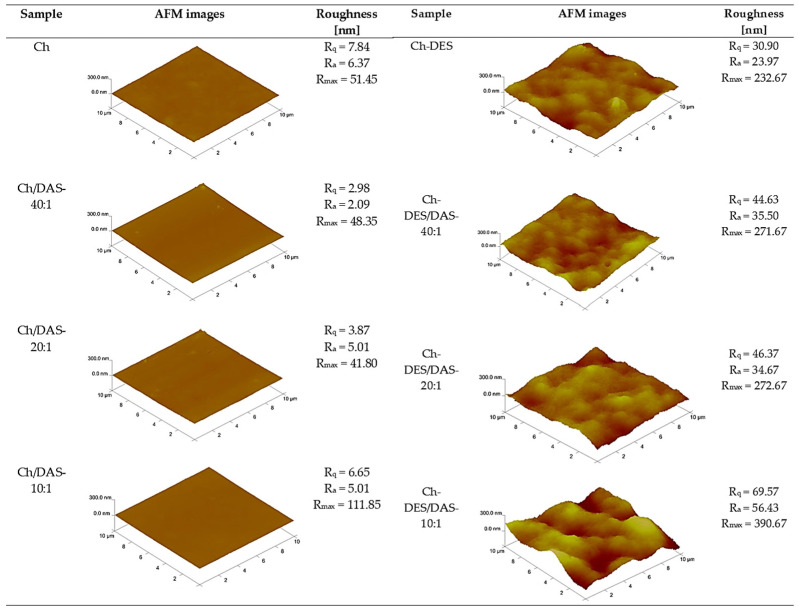
AFM images of Ch, Ch-DES, Ch/DAS, and Ch-DES/DAS films.

It is well known that AFM analysis enables the assessment of the homogeneity of polymer blends, which affects not only their structure but also the functional properties of the resulting materials. Figure 5 presents AFM images of the tested samples together with the Rq, Ra, and Rmax values. The meaning of these parameters has been discussed in detail in our previous papers [22,30].

Introducing different amounts of dialdehyde starch into chitosan matrix leads to only slight changes in the roughness parameters, indicating that the obtained systems are compatible and yield a smooth surface free of cracks and inhomogeneities. Such a behavior can be an effect of structural similarity between both film components.

In contrast, the addition of a deep eutectic solvent based on choline chloride and malonic acid promotes the formation of wrinkles and surface irregularities during film formation. The increase in roughness is evident for both chitosan-based materials containing varying amounts of dialdehyde starch and for systems that incorporate DESs as well. As demonstrated in our previous work [22], the introduction of a DES composed of choline chloride and malonic acid disrupts inter- and intramolecular hydrogen bonds, as also given in FTIR-analysis section, which affects the surface topography. In systems containing an additional polymer in the form of dialdehyde starch, responsible for forming a covalent network between chitosan and starch derivative, the distribution of plasticizer molecules is hindered. As a result, the roughness increases with increasing starch content in the studied materials.

### 3.5. Modification of Mechanical Properties

It is well known that films based on pure chitosan exhibit poor mechanical properties. They are stiff and brittle, which significantly limits their application possibilities. Therefore, chitosan-based materials are subjected to various modifications to enhance their applicability. Depending on the potential application, chitosan is subjected to crosslinking processes using compounds such as genipin, dialdehyde starch, D-fructose, tannic acid, or glutaraldehyde [51,52,53,54,55,56], or plasticizers are introduced in the form of deep eutectic solvent, epoxidized soybean oil, and epoxidized palm oil, as well as glycerol, PEG-400, and sorbitol [21,57,58,59].

To improve the properties of chitosan in this work, both a crosslinking agent in the form of dialdehyde starch (DAS) and a plasticizer in the form of a deep eutectic solvent consisting of choline chloride and malonic acid (DES) were introduced.

**Figure 6:**
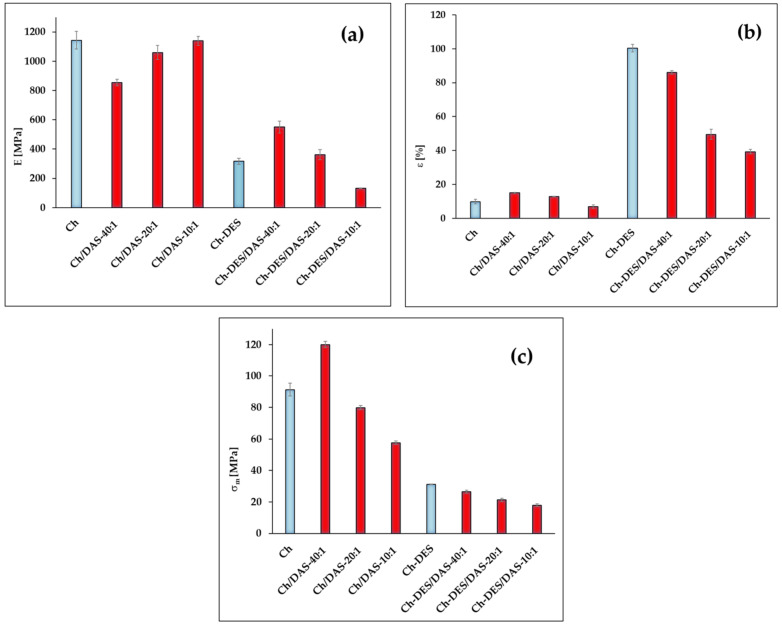
Mechanical properties of studied materials: (**a**) Young’s modulus, (**b**) elongation at break, (**c**) tensile strength.

Figure 6a shows the Young’s modulus values calculated based on the obtained stress–strain curves. It was observed that the addition of DAS, regardless of the amount introduced, lowers the Young’s modulus.

It should be emphasized that with small amounts (40:1 ratio), the stiffness of the formed material significantly decreased, which may indicate uneven distribution of crosslinking bonds in the structure of the obtained material. In the case of introducing larger amounts of dialdehyde starch, the structure becomes stiffer, leading to an increase in Young’s modulus; however, the crosslinked materials ultimately do not reach the Young’s modulus value obtained for pure chitosan. Most scientific papers indicate that adding a crosslinking agent to chitosan results in a significant increase in Young’s modulus [60]. However, there are also papers that prove crosslinked chitosan has significantly lower E values than pure chitosan [61,62,63]. The authors of these papers suggest that in this case, chitosan undergoes excessive crosslinking, leading to brittleness rather than reinforcement.

To mitigate the excessive brittleness of the material, a deep eutectic solvent was introduced into the above-described systems, which in this case acts as a plasticizer. Figure 6a clearly shows that introducing DESs into pure chitosan lowers the Young’s modulus value several times; a similar effect is observed in the case of the materials containing the smallest amounts of DAS. On the other hand, adding DESs to systems where the crosslinking agent is not present in large amounts (20:1 and 40:1) results in a compromise between crosslinking and plasticization of the system. Namely, there is an increase in Young’s modulus compared to the chitosan–deep eutectic solvent system, but the materials are not as stiff and brittle as in the case of pure chitosan or chitosan with the addition of only the crosslinking agent in the form of dialdehyde starch. Based on the literature, it can be assumed that the addition of DESs breaks and shields the hydrogen bonds present in chitosan, increasing the mobility of polymer chains and leading to the formation of flexible films [22,30], as confirmed by the elongation at break values shown in Figure 6b.

It is well known that chitosan is a stiff and brittle polymer, so the elongation at break does not exceed 10%. Such a film is stiff, with relatively high strength, but breaks with little deformation, which limits its usefulness as a highly flexible packaging material. A similar situation occurs when a crosslinking agent, in the form of dialdehyde starch, is introduced. The crosslinked material appears more durable, albeit slightly, and the values for these materials do not exceed 13%, indicating a dominant crosslinking effect: Schiff base bonds form between the aldehyde groups of DAS and the amino groups of chitosan [64,65]. On the other hand, after introducing DESs into the systems, a competitive effect of crosslinking by DAS and plasticization by DESs is clearly observed.

In the case of all ternary systems, a clear decrease in elongation at break is observed in comparison to sample Ch-DES, suggesting that crosslinking by DAS partially cancels the plasticizing effect of DESs, resulting in a denser network and limited chain mobility [66]. Nevertheless, the elongation at break is still several times higher than in samples without DES addition, indicating that DES remains the dominant plasticizer, while DAS only provides some degree of system stiffness to a certain extent.

A surprising effect was achieved for the Ch-DES/DAS-10:1 sample, where the elongation at break is about 86%, which is only slightly lower than for the Ch-DES material, suggesting that at this ratio of crosslinking agent to plasticizer, a certain compromise between crosslinking and plasticization is observed, namely, the structure is slightly stiffened but not to the extent that it reduces the mobility of chitosan segments caused by DES.

The literature has shown [67] that at an appropriate level of crosslinking, strength, and stability can be improved with only a slight decrease in elongation, suggesting that the area around a ratio close to 10:1 is potentially the best combination of mechanical properties for the studied systems.

Meanwhile, the data obtained for tensile strength (Figure 6c) confirm the above-described effects. Namely, the introduction of DAS at a higher ratio causes a decrease in tensile strength by forming a denser network of Schiff base bonds, thereby increasing the material’s brittleness, which is confirmed by the literature [68].

The Ch-DES material exhibits a drastic drop in tensile strength, from 91 MPa to 31 MPa, which is a standard effect of DES action. As DES increases chain mobility, it weakens hydrogen bonds and reduces tensile strength by 50–70%, as indicated in the literature [69]. Surprisingly, in the ternary systems, no significant increase in tensile strength values is observed compared to the Ch-DES sample. This is probably due to the dominant plasticizing effect of DESs, which permanently weakens the interchain interactions of chitosan, preventing full strength recovery by the crosslinking agent in the form of DAS [64].

In summary, the obtained results suggest that, in terms of mechanical properties, the most promising material is the ternary system with the highest ratio of amino groups to aldehyde groups, at 10:1.

### 3.6. Changes in Color Parameters

The colors of polymer packaging films play a significant role in influencing consumer perception of products. Packaging color attracts customer attention and influences product attractiveness. However, the need to prioritize sustainable and environmentally friendly packaging solutions is increasingly emphasized. Such alternatives may exhibit slight esthetic imperfections, but their ecological advantages often outweigh these minor shortcomings [70].

In this study, the CIELab method was employed to assess the impact of DAS cross-linking and/or deep eutectic solvent plasticizer on the material’s colors.

In Table 2, the obtained data are presented in the form of L, a, and b values, as well as the total color difference (ΔE), chroma (C), yellowness index (YI), and browning index (BI) values calculated based on the aforementioned formulas (Equations (3)–(6)). Before the discussion of the obtained results, it should be stressed that color difference ΔE greater than two units is generally considered visibly perceptible to the average observer, whereas values above 5 indicate a clearly distinguishable color [71,72].

Based on the calculated ΔE, it is evident that both additives have a significant influence on the color changes in the studied materials. CIELab results indicate noticeable differences in film color after DAS modification. As the dialdehyde starch content in the material increases, the values of all calculated parameters rise, indicating that the addition of this crosslinking agent significantly affects the visual appearance of the packaging, causing it to be noticeably yellow. The mentioned above effect is caused by the formation of conjugated Schiff base chromophores [73].

Introducing a deep eutectic solvent into chitosan, in the form of a mixture of choline chloride and malonic acid, also affects the film’s color change, as well as the values of other parameters, such as chroma and yellowness. However, these changes are not as significant as in the case of adding dialdehyde starch alone. On the other hand, an interesting effect on the analyzed parameters is observed in ternary systems. Namely, the addition of a small amount of dialdehyde starch to the Ch-DES system causes a significant increase in ΔE, C, and YI values compared to the material without DESs. Whereas in the case of materials with increased DAS content (20:1 and 10:1), no significant changes are observed compared to materials consisting of chitosan with the addition of the crosslinking agent. Both color changes, as well as chroma and yellowness for these samples, remain at nearly unchanged levels. This may suggest that the higher content of dialdehyde starch balances the changes caused by the presence of DESs.

The situation is different in the case of browning (Figure S3, Supplementary Materials). The literature describes that chitosan crosslinked with dialdehyde starch browns primarily through Maillard-like reactions between the reducing sugar ends in DAS and the amino groups in chitosan, forming melanoidins, which are brown pigments [74]. It should be emphasized that higher DAS doses accelerate this process, which correlates with the presented results (Table 2). Additionally, Schiff base chromophores, initially appearing yellow, are oxidized to melanoidin structures, which further promote browning [73].

Moreover, it is clear that DES also influences changes in the color of the materials being studied. It has been demonstrated in various publications that DESs can intensify browning in chitosan-DAS films by accelerating Maillard reactions through its acidic nature [75]. It was established that the system, consisting of choline chloride and malonic acid, lowers the pH, promoting the Amadori rearrangement to melanoidins, and enhances molecular mobility, which facilitates interactions between DAS reducing groups and chitosan amines [76,77].

### 3.7. Wettability and Surface Free Energy

The mean values of water and diiodomethane contact angle on all the tested surfaces are provided in Table 3, along with the surface free energy (SFE) values and their polar and dispersive components.

The relatively high water contact angle of H_2_O and the contact angle of CH_2_I_2_ on the chitosan film surface below 90° suggest the surface hydrophobic nature, which does not correspond to the molecular structure of the chitosan macromolecule. A similar behavior has been observed by Pavoni et al. [78] and by Tanpichai et al. [54] for chitosan films cast from acetic acid solution. The hydrophobic surface behavior can be attributed to the reorganization of macromolecules on the liquid–air interface during drying, reflected by the orientation of chitosan functional groups (–OH/–NH_2_) more parallel to the film surface, which reduces the effective density of polar sites presented to the droplet. As discussed by Tanpichai et al. [54], the water contact angle decreases with time as the water droplet stays on the surface, and the higher water CA values are characteristic of measurements taken at early times (5s in the current studies). The calculated surface free energy values and their components, especially the highly dispersive ones, are in agreement with contact angle measurements and previously reported SFE values obtained for chitosan films prepared using the same method and glycerine/diiodomethane set as the tested liquids [79].

It is well known that the apparent wettability is strongly influenced by surface topography, particularly surface roughness. As demonstrated by AFM measurements (Figure 5), all Ch/DAS films are smooth and do not vary substantially from neat chitosan; therefore, no topography effect should be considered. Thus, it can be stated that the changes observed in the hydrophilic/hydrophobic nature of the dialdehyde-crosslinked chitosan film surface can be an effect of two competing surface effects [37,80]: (i) the introduction of hydroxyl-rich starch segments (potentially increasing water affinity), and (ii) the consumption of chitosan –NH_2_ sites into crosslinks (often reducing the effective polar contribution accessible to probe liquids). As can be seen in Table 3, the water contact angle for Ch/DAS films decreases, and the CH_2_I_2_ contact angle increases monotonically as DAS content increases. That pattern indicates a progressive increase in apparent water wettability and hydrophilicity of the tested surfaces. Such behavior suggests the enrichment exposure of hydrophilic Ch and DAS functional groups at the air-facing surface, as also suggested by others [37]. It can also be noted that the addition of DAS first causes a slight increase in $\gamma_{S}$ to 36.41 mJ·m^−2^, and then the total surface free energy gradually decreases as DAS content rises, to 29.64 mJ·m^−2^ at the highest DAS content. A similar behavior has been given by Węgrzynowska-Drzymalska et al. [65]; however, the authors did not note any particular trend when different amounts of the crosslinkers were applied. Simultaneously with the monotonic changes in SFE, a gradual decrease in both polar and dispersive components was found. For the Ch/DAS-10:1 film, the $\gamma_{S}^{P}$ reaches only 0.02 mJ·m^−2^. These results contradict the previously mentioned literature data [65]. The variation can be a result of a different set of tested liquids (CH_2_I_2_/H_2_O vs. CH_2_I_2_/glycerin). The problem of comparing and varying the SFE data obtained with different liquids has already been discussed in the literature [81]. Moreover, in the case of chitosan-based surfaces and polar liquids, a time-dependent decrease in contact angle values was observed by Marton et al. [82]. Thus, measurements should be taken at a given and relatively short time after droplet creation, as was performed in the current work, to prevent surface changes caused by swelling. The decrease in the polar component with increasing DAS content can also be attributed to amine consumption during the crosslinking reaction.

The addition of DESs (50 wt.% content) causes a substantial decrease in the water contact angle from 120.1° ± 2.8° (Ch) to 96.6° ± 2.8° (Ch-DES). At this same time, the diiodomethane contact angle increases only by 1.6°, suggesting that the nonpolar/dispersive interaction balance at the outermost surface is comparatively less affected than the water-facing (acid–base/polar) interactions. Simultaneously, even if substantial differences in water wetting behavior, the polar surface free energy component stays almost unaffected. AFM results (Figure 5) indicate a large roughness increase after DES addition (e.g., Rq increases from 7.84 nm (Ch) to 30.90 nm (Ch-DES)) and demonstrate a more substantial topographically heterogeneous surface after introduction of plasticizer. Similar roughness changes have already been discussed by us earlier [30]. Classical wetting theory predicts that roughness can cause a shift in measured θ values, even at a fixed surface chemistry [83]. Therefore, the fact that water contact angle decreases despite a strong increase in roughness implies that interfacial changes caused by the DES dominate over the geometric ones (the DES effect is not merely morphological but also modifies water–surface interactions). As the choline-chloride/malonic acid mixture is known to act as a chitosan plasticizer, it increases chain mobility and promotes rearrangements during drying, resulting not only in rougher microdomains but also in changes to the previously discussed reorganization of macromolecules at the liquid-air interface during drying. Finally, improved viability of –OH/–NH_2_ functionals on the film’s surface is obtained. Moreover, the hydrophilic nature of DES itself should not be overlooked.

The introduction of DAS into Ch-DES drives a strong, monotonic decrease in water contact angle from 96.6° for Ch-DES to 60.5°, 56.2°, and 45.5° for Ch-DES/DAS films with increasing DAS content, respectively. The changes correspond with the progressive increase in the hydrophilic nature of the outermost film surface layer. Moreover, a very large rise in the polar component $\gamma_{S}^{P}$ of surface free energy after adding DAS (from 0.82 to ca.18–22 mJ·m^−2^) was also found. It is worth emphasizing that the enhancement of polarity is the opposite effect noted for neat chitosan systems crosslinked with the same amount of DAS. Simultaneously to these changes, the diiodomethane contact angle shows the opposite overall trend to the neat DAS-crosslinked chitosan films, i.e., after an initial increase at the lowest DAS loading (~64.2° at 40:1), it decreases substantially at higher DAS (to 55.8° at 20:1 and 45.6° at 10:1 NH_2_:CHO ratios), indicating enhanced wetting by a non-polar liquid at higher crosslinker content. The decreasing diiodomethane contact angle is consistent with an increase in the effective dispersive interaction term at the surface (Table 3), reflected by changes of $\gamma_{S}^{D}$ values from 26.2 for Ch-DES/DAS-40:1 to 36.7 mJ m^−2^ for Ch-DES/DAS-10:1.

The changes observed for DAS-crosslinked Ch-DES samples can be explained by the plasticizer effect that causes a larger fraction of the polysaccharide-rich (chitosan/dialdehyde starch) network to be exposed at the interface and/or because the surface becomes topographically rougher, amplifying wetting. According to the previously reported Wenzel regime (for liquid fully following the texture), increasing roughness amplifies the intrinsic wetting tendency: for intrinsically wetting surfaces (θ < 90°), roughness lowers the apparent contact angle, whereas for non-wetting surfaces it increases it [83,84]. After DAS addition, water angles are all < 90°, so the observed progressive decrease in water contact angle with increasing DAS is fully compatible with a combined effect of (i) higher surface polarity and (ii) roughness-driven amplification of hydrophilicity.

### 3.8. UVA and UVB Resistance

**Figure 7:**
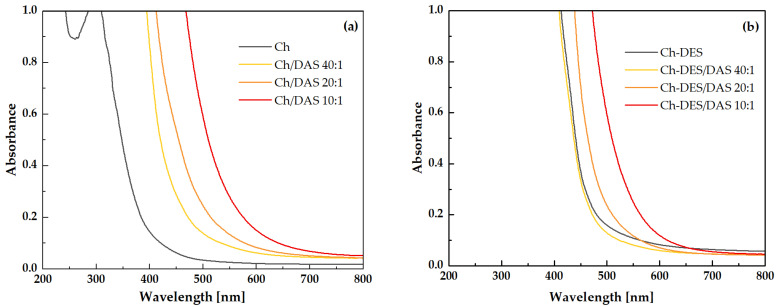
UV-Vis spectra of (**a**) chitosan and chitosan crosslinked with DAS, (**b**) materials containing DESs.

Thanks to the UVA and UVB barrier properties of packaging materials, the food products are protected against photodegradation, oxidation, and loss of nutritional value. It is well known that UVA and UVB radiation induce photochemical damage in food, including the degradation of vitamins (e.g., A, C, D), pigments, lipids, and riboflavin, thereby shortening shelf life [85,86]. Figure 7a shows the UV-Vis spectra for pure chitosan and chitosan cross-linked with DAS, while Figure 7b shows the spectra obtained for materials containing DESs.

**Figure 8:**
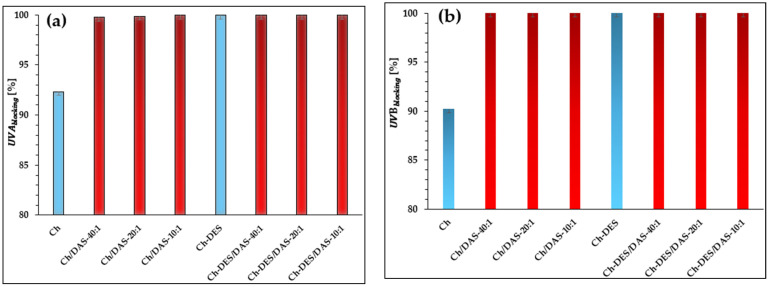
Resistance of the tested materials to (**a**) UVA and (**b**) UVB.

Based on the obtained data, the UVA and UVB barrier properties of the investigated systems were calculated. The charts depicting the barrier values for the prepared films are presented in Figure 8a,b.

Neat chitosan already exhibits excellent protective properties against UVA and UVB radiation, attributable to its chemical structure and dense film morphology. Chitosan contains amino and hydroxyl groups, along with residual N-acetylglucosamine units, which form chromophores absorbing in the near-ultraviolet region (ca. 200–280 nm). Furthermore, a portion of the radiation is scattered at the boundaries between amorphous and crystalline domains, further enhancing the UVA and UVB barrier performance [85]. The addition of dialdehyde starch (DAS) alters the structure, inducing crosslinking in the chitosan-based material and thereby increasing material density and strengthening UV barrier properties, as confirmed by Yong et al. [73].

The incorporation of DES, composed of malonic acid and choline chloride, into chitosan induces hydrogen-bonding interactions and introduces functional groups (C=O, OH, Cl^−^) derived from its components, thereby reducing UVA and UVB transmittance [76]. However, it should be noted that the addition of both DAS and DES to chitosan does not alter the absorption spectra. This is likely attributable to structural modifications in the materials, including a reduced crystalline phase content and lower film density, which collectively result in no significant enhancement of UVA and UVB resistance compared to DAS-crosslinked chitosan films alone [87].

## 4. Conclusions

Based on the obtained results, it was established that chitosan-based films modified with dialdehyde starch (DAS) and a choline chloride–malonic acid deep eutectic solvent (DES) form materials with tunable physicochemical properties, making them potentially suitable for packaging applications. The incorporation of DES, composed of choline chloride and malonic acid, into chitosan crosslinked with dialdehyde starch (DAS) is crucial, as it acts as an efficient plasticizer that counterbalances the crosslinking effects induced by DAS on chitosan. Analysis of individual formulations revealed that the Ch-DES/DAS-40:1 film represents the most promising material. It exhibits reduced density (1.327 g·cm^−3^) compared to pure chitosan (1.403 g·cm^−3^) and crosslinked chitosan (1.343 g·cm^−3^). Furthermore, the incorporation of both modifiers preserved satisfactory material stiffness (Young’s modulus of 550 MPa) while ensuring substantial flexibility (elongation at break ≈90%), which is particularly relevant for prospective applications. Additionally, the specified sample achieved a surface free energy of 44.38 mJ·m^−2^, indicating moderately high surface hydrophilicity, which may be advantageous for active food packaging applications. Overall, combining DAS crosslinking with DES plasticization enables deliberate balancing of stiffness, flexibility, and hydrophilicity, providing a rational approach to tailor chitosan-based films for advanced, multifunctional biodegradable packaging. The potential incorporation of antibacterial or antioxidant compounds into the developed system could broaden the application scope of these materials and uncover their capacity to extend the shelf life of food products.

## Figures and Tables

**Table 1:** Thickness (*h*) and density (*d*) of chitosan-based films.

Sample	*h* [mm]	*d* [g·cm^−3^]
Ch	0.237 ± 0.043	1.403
Ch/DAS-40:1	0.238 ± 0.032	1.343
Ch/DAS-20:1	0.245 ± 0.015	1.401
Ch/DAS-10:1	0.244 ± 0.013	1.442
Ch-DES	0.239 ± 0.022	1.264
Ch-DES/DAS-40:1	0.243 ± 0.067	1.327
Ch-DES/DAS-20:1	0.233 ± 0.054	1.331
Ch-DES/DAS-10:1	0.270 ± 0.062	1.214

**Table 2:** Color parameters of neat, DAS-crosslinked, and/or DES-plasticized chitosan films.

Sample	*L*	*a*	*b*	Δ*E*	*C*	*YI*	*BI*
Ch	87.8 ± 0.2	−0.2 ± 0.1	−2.6 ± 0.2	−	2.6	−4.2	−3.0
Ch/DAS-40:1	82.5 ± 0.2	−1.0 ± 0.1	16.2 ± 0.8	19.5	16.2	28.0	20.1
Ch/DAS-20:1	74.2 ± 0.8	4.0 ± 0.5	35.8 ± 0.7	40.9	36.0	68.9	61.7
Ch/DAS-10:1	73.6 ± 0.5	7.7 ± 0.4	41.0 ± 0.1	46.5	41.7	79.5	84.4
Ch-DES	85.1 ± 0.3	−1.6 ± 0.2	7.1 ± 0.5	10.0	7.0	11.5	6.7
Ch-DES/DAS-40:1	82.0 ± 2.5	−3.6 ± 0.8	24.6 ± 2.1	26.1	22.9	39.4	27.7
Ch-DES/DAS-20:1	75.9 ± 0.8	0.7 ± 0.1	36.0 ± 0.2	40.5	36.0	67.8	66.4
Ch-DES/DAS-10:1	64.4 ± 2.7	13.8 ± 3.3	35.2 ± 1.4	46.7	37.8	78.2	90.9

**Table 3:** Surface free energy ($\mathrm{SFE},\;\gamma_{S}$), dispersive ($\gamma_{S}^{D}$), and polar ($\gamma_{S}^{P}$) components calculated for the native, plasticized, and DAS-crosslinked chitosan films.

Sample	Average Contact Angle [°]		Surface Free Energy [mJ·m^−2^]		
	Water	Diiodomethane	$\gamma_{S}$	$\gamma_{S}^{D}$	$\gamma_{S}^{P}$
Ch	120.1 ± 2.8	55.3 ± 2.7	32.53	31.29	1.24
Ch/DAS-40:1	117.1 ± 3.1	48.4 ± 2.2	36.41	35.19	1.22
Ch/DAS-20:1	109.4 ± 4.2	53.1 ± 3.2	32.66	32.55	0.11
Ch/DAS-10:1	105.8 ± 0.2	58.2 ± 2.2	29.64	29.62	0.02
Ch-DES	96.6 ± 2.8	56.9 ± 3.9	31.17	30.35	0.82
Ch-DES/DAS-40:1	60.5 ± 2.5	64.2 ± 2.5	44.38	26.19	18.19
Ch-DES/DAS-20:1	56.2 ± 1.4	55.8 ± 1.6	49.40	31.02	18.38
Ch-DES/DAS-10:1	45.5 ± 2.0	45.6 ± 2.2	58.91	36.72	22.20

## Data Availability

The original contributions presented in the study are included in the article/Supplementary Materials. Further inquiries can be directed to the corresponding authors.

## Supplementary Materials

The following supporting information can be downloaded at https://www.mdpi.com/article/10.3390/ma19030529/s1. Figure S1: FTIR spectra of neat chitosan film (Ch) and Ch films crosslinked with dialdehyde starch of different crosslinker content. Figure S2: FTIR spectra of choline chloride:malonic acid deep eutectic solvent. Figure S3: Films’ image taken on a black background.
