# Supplementary material for: Green Deep Eutectic Solvents for Functionalizing Chitosan–Dialdehyde Materials with Varied Crosslinker Content
Source: Materials (Basel). 2026 Jan 29;19(3):529. doi: 10.3390/ma19030529 (PMC12897872; doi:10.3390/ma19030529)
Supplement: Supplementary file 1 [file materials-19-00529-s001.zip › Supplementary materials_FigureS1.pdf]

Supplementary Materials

Green deep eutectic solvents for functionalizing chitosan–dialdehyde materials with varied crosslinker content

Magdalena Gierszewska [1], Ewa Olewnik-Kruszkowska [1,*], Kornelia Kadac-Czapska [2,*], Małgorzata Grembecka [2] and Eliza Knez [2]

1 Department of Physical Chemistry and Physicochemistry of Polymers, Faculty of Chemistry, Nicolaus Copernicus University in Toruń, 87-100 Toruń, Poland;
2 Department of Bromatology, Faculty of Pharmacy, Medical University of Gdańsk, 80-416 Gdańsk, Poland; e-mail@e-mail.com
* Correspondence: olewnik@umk.pl (E.O.-K.); kornelia.kadac@gumed.edu.pl (K.K.-Cz.)

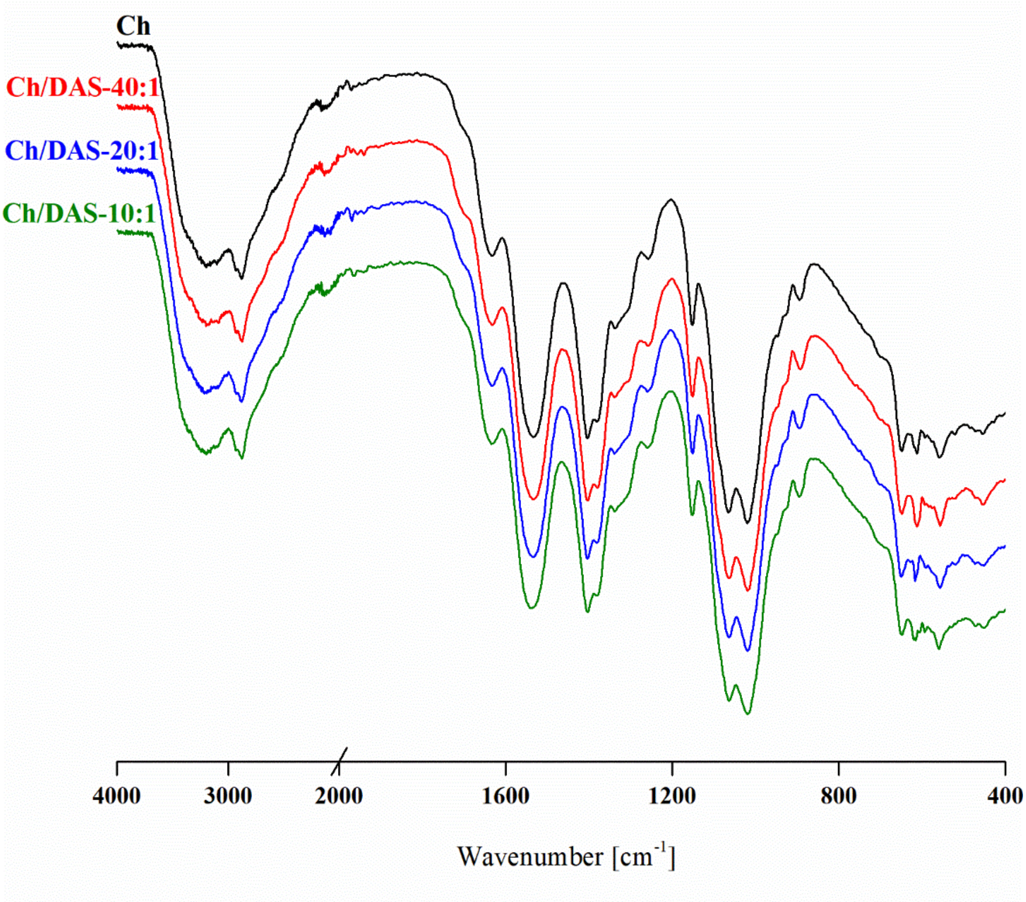

Figure S1. FTIR spectra of neat chitosan film (Ch) and Ch films crosslinked with dialdehyde starch of different crosslinker content.
